# Post-infectious psychiatric symptoms and diagnoses in children and adolescents following COVID-19

**DOI:** 10.1186/s12888-026-08217-4

**Published:** 2026-05-23

**Authors:** Mehmet Cengiz, Şefika Nurhüda Karaca Cengiz, Ayşe Büyükcam, Ahmet Bolat, Bülent Ünay, Yasemin Taş Torun

**Affiliations:** 1 Department of Pediatrics, Özel Gürlife Hospital, Akınsel Street, No: 1, Tepebaşı, Eskişehir, Türkiye; 2 Department of Child and Adolescent Mental Health and Disease, Eskişehir City Hospital, Eskişehir, Türkiye; 3 Division of Pediatric Infectious Diseases, Department of Pediatrics, Istanbul Faculty of Medicine, Istanbul University, Istanbul, Türkiye; 4 Department of Pediatrics, University of Health Sciences Gülhane Medical Faculty, Ankara, Türkiye; 5 Division of Pediatric Neurology, Department of Pediatrics, Gülhane Faculty of Medicine, University of Health Sciences, Ankara, Türkiye; 6 Department of Child and Adolescent Mental Health and Disease, Faculty of Medicine, Gazi University, Ankara, Türkiye

**Keywords:** COVID-19, Children, Adolescents, Anxiety, Depression, RCADS, Mental health, Psychiatric symptoms

## Abstract

**Background:**

The Coronavirus Disease 2019 (COVID-19) pandemic has substantially disrupted the lives of children and adolescents, raising concerns about increased psychiatric morbidity. This study examined post-COVID psychiatric symptoms in a pediatric population and explored associated risk factors.

**Methods:**

This follow-up study enrolled 107 children and adolescents (6–18 years) with laboratory-confirmed COVID-19, retrospectively identified from Gülhane Training and Research Hospital records, contacted via telephone, and invited for in-person pediatric examination and Revised Child Anxiety and Depression Scale (RCADS) screening. Participants with T-scores ≥65 were referred to Gazi University for face-to-face psychiatric evaluation by child psychiatrists using DSM-5 criteria. Sociodemographic and clinical variables were analyzed for associations with elevated RCADS scores. Receiver operating characteristic (ROC) analyses were performed using DSM-5–based clinical diagnoses as the reference standard.

**Results:**

The cohort included 62 females (57.9%) and 45 males (42.1%), with a mean age of 12.3±3.1 years. Most participants were symptomatic during infection (90.7%), and 35.5% developed at least one COVID-related complication, most commonly taste loss (29.0%). Post-COVID psychiatric symptoms were reported in 56.1% of cases, predominantly attention difficulties (28.9%) and irritability (23.3%). Approximately half (52.2%) of children without prior history of psychiatric symptoms developed at least one new symptom. Higher anxiety and depression scores were significantly associated with prematurity, birth complications, and pre-existing psychiatric symptoms. In ROC analyses, the parent-reported total anxiety score showed an area under the curve (AUC) of 0.92 with an optimal cut-off of 60 (sensitivity 81.5%, specificity 93.8%), while the child-reported score showed an AUC of 0.90 with an optimal cut-off of 65 (sensitivity 70.4%, specificity 93.8%). Children without psychiatric history had significantly lower scores (p < 0.001).

**Conclusions:**

COVID-19 was linked to increased psychiatric symptoms in children, with prematurity, birth complications, and prior history as key risks. RCADS proved sensitive for screening, emphasizing targeted interventions post-infection.

**Clinical trial number:**

Not applicable.

## Background

The Coronavirus Disease 2019 (COVID-19) first emerged in Wuhan, China, in December 2019 and quickly spread worldwide, prompting the World Health Organization (WHO) to declare it a global pandemic on March 11, 2020 [6, 27]. Despite the progression of time and the availability of vaccines and treatments, the long-term physical and psychological consequences of the pandemic continue to unfold [3].

Children and adolescents were significantly affected by the societal disruptions caused by the pandemic. School closures, the transition to remote learning, and the loss of daily routines severely impacted their academic, social, and emotional development [18]. Families faced increased stress levels due to isolation, economic uncertainty, and disrupted routines, often resulting in heightened familial conflict and altered dynamics [25]. Moreover, access to psychiatric care became limited, particularly for children and adolescents with pre-existing mental health conditions, leading to further exacerbation of symptoms [11].

Emerging evidence suggests that children and adolescents exposed to COVID-19, either directly through infection or indirectly through pandemic-related disruptions, are at elevated risk for a range of psychiatric symptoms. In adult populations infected with COVID-19 reported prevalence rates among infected individuals include 54.5% for post-traumatic stress disorder (PTSD), 39% for depression, 32.5% for panic disorder, and 15.6% for obsessive-compulsive disorder [16]. However, most of these data stem from adult populations, and there remains a paucity of research focusing specifically on pediatric and adolescent cohorts [24].

Understanding the long-term mental health implications of COVID-19 in younger populations is critical for informing the development of targeted interventions that promote resilience and psychological well-being [22].

This study aimed to assess post-COVID psychiatric symptoms in children/adolescents via telephone follow-up and structured screening, identifying risk factors for vulnerability.

## Methods

### Study design and participants

This study was conducted as a single-center follow-up cohort involving pediatric patients with a prior laboratory-confirmed Severe Acute Respiratory Syndrome Coronavirus 2 (SARS-CoV-2) infection. Children and adolescents aged 6–18 years were retrospectively identified from hospital PCR records at the Pediatric Infectious Diseases Department of the University of Health Sciences, Gülhane Training and Research Hospital (Ankara, Türkiye). Eligible patients were contacted by the study team and invited to attend a face-to-face follow-up visit between January 1, 2021, and January 1, 2022, after recovery from COVID-19. While participants were identified and contacted through the Gülhane hospital system, face-to-face psychiatric evaluations were conducted at Gazi University Faculty of Medicine, Department of Child and Adolescent Psychiatry.

During the follow-up visit, participants underwent a standardized pediatric examination and completed questionnaire-based mental health screening. All participants were administered the Revised Child Anxiety and Depression Scale (RCADS), including both child self-report and parent-report forms, as part of the screening protocol. All parent rating scales were completed by the primary caregivers, who were the biological mothers of the participants Participants with RCADS T-scores ≥ 65 on either form were referred for in-person psychiatric evaluation conducted by child and adolescent psychiatrists in accordance with DSM-5 criteria at Gazi University Faculty of Medicine, Department of Child and Adolescent Psychiatry.

Written informed consent and assent were obtained from all caregivers and children prior to participation. A developmentally appropriate informed consent/assent form was used and completed with parental support when needed. The study protocol was approved by the Institutional Ethics Committee of Gazi University Faculty of Medicine (approval number: 2022 − 167), and was conducted in accordance with the Declaration of Helsinki. No a priori sample size calculation was performed, as the sample size was determined by the number of eligible patients who attended the follow-up visit during the study period.

### Inclusion and exclusion criteria

**Inclusion criteria included**:

- Age between 6 and 18 years.
- Laboratory-confirmed history of COVID-19.
- Completed mental health screening using both RCADS-Parent and RCADS-Child forms.

**Exclusion criteria were**:

- Presence of sensory impairments (e.g., severe vision or hearing loss) interfering with questionnaire completion.
- Inability to read or write.
- Intellectual disability that precluded self-report assessment.
- Missing or incomplete informed consent.

## Measures

### Revised child anxiety and depression scale (RCADS)

The RCADS is a validated 47-item scale developed by Chorpita et al. (2000) [5] for assessing symptoms of anxiety and depression in children and adolescents. It includes six subscales: Major Depressive Disorder (MDD), Generalized Anxiety Disorder (GAD), Panic Disorder, Separation Anxiety Disorder, Social Phobia, and Obsessive–Compulsive Disorder. Both the child version (self-report) and parent version were used in this study. Scoring was performed using the RCADS Scoring Program (Version 3.2, Excel-based), and a T-score of 65 or higher was considered clinically significant [8].

### Data collection and analysis

All assessments were conducted in clinical settings by trained personnel. Psychiatric diagnoses were established through face-to-face clinical evaluation by child and adolescent psychiatrists in accordance with DSM-5 criteria. Demographic and clinical data, including prior psychiatric history, COVID-19-related clinical course, and family mental health history, were obtained from medical records and structured parent interviews.

Statistical analyses were performed using IBM SPSS Statistics Version 30.0 for Mac (IBM Inc., Armonk, NY, USA). Descriptive statistics were used to summarize demographic and clinical characteristics. Categorical variables were compared using *Chi-square tests*, while continuous variables were compared using the *Student’s t-test* or *Mann–Whitney U test*, depending on the normality of distribution (as assessed by the Shapiro–Wilk test). A p-value of < 0.05 was considered statistically significant.

## Results

A total of 107 children and adolescents (62 females [57.9%], 45 males [42.1%]) with a mean age of 12.3 ± 3.1 years (range: 6–17 years) were included in the study. Seventeen participants (15.9%) had a history of chronic medical conditions, including asthma (*n* = 4), beta-thalassemia (*n* = 2), hypothyroidism (*n* = 2), and one case each of allergy, ataxia, bicuspid aortic valve, eczema, familial mediterranean fever, hypertension, valvular heart disease, and vesicoureteral reflux. All participants had completed age-appropriate routine vaccinations.

Hospitalization history prior to COVID-19 was reported in 42 patients (39.3%), while 23 (21.5%) had undergone at least one surgical intervention. Most participants were born at term (88.8%), with 10.3% born prematurely and 0.9% post-term. Birth complications were reported in 5 participants (4.7%), including respiratory distress (*n* = 3) and neonatal jaundice (*n* = 2).

Chronic illness in first-degree relatives was present in 66.4% of the participants’ families. Cardiovascular diseases (32.7%), endocrine disorders (19.6%), malignancies (6.5%), and other chronic conditions (7.5%) were the most commonly reported. Notably, 29% had more than one chronic illness within the family.

Family history of COVID-19 infection was reported in 92 participants (86.0%), most commonly among nuclear family members (64.5%), followed by mothers (11.2%), fathers (3.7%), and siblings (3.7%).

*At the time of presentation*, 97 participants (90.7%) were symptomatic, reporting a mean of 2.9 ± 1.8 symptoms (range: 0–6). The most frequently reported symptoms included fever (62.6%), cough (10.3%), myalgia (4.7%), anosmia and ageusia (4.7%), rhinorrhea (3.7%), headache (1.9%), sore throat (1.9%), and dyspnea (0.9%). Fever was the most common symptom in cases with multiple complaints, often co-occurring with cough and myalgia. *All participants were evaluated in the pediatric emergency department or outpatient clinics and were managed on an outpatient basis; none required hospitalization due to COVID-19 infection*.

All COVID-19 diagnoses were confirmed via PCR testing, performed an average of 1.2 ± 0.5 times (range: 1–4). The majority of participants (97.2%) were monitored at home for 7–14 days, while only three (2.8%) had shorter isolation periods. On average, patients received 2 (± 0.1) medications for COVID-19.

COVID-19-related complications were observed in 38 patients (35.5%), with taste loss being the most frequent (*n* = 31, 29.0%). Olfactory dysfunction and pulmonary complications were each observed in three patients. One participant developed an additional unspecified complication. Among patients with taste loss, most also reported anosmia.

Notably, 15% of the participants had experienced a COVID-related death among relatives, most commonly grandparents, paternal aunts, and uncles. Reinfection with COVID-19 was reported in 7.5% of the cohort. Among participants with available variant data, the Delta variant was the most frequently recorded, while the remaining cases were classified as unspecified or other variants. Table 1 summarizes demographic characteristics of participants.

**Table 1:** Demographic and clinical characteristics of participants

Variable	*n* (%)
**Gender**	
Female	62 (57.9%)
Male	45 (42.1%)
**Mean Age (years)**	12.3 ± 3.1
**Chronic Medical Conditions**	17 (15.9%)
Asthma	4
Beta-thalassemia	2
Hypothyroidism	2
Other (1 case each)	9
**Prior Hospitalization**	42 (39.3%)
**History of Surgery**	23 (21.5%)
**Gestational Age**	
Term	95 (88.8%)
Preterm	11 (10.3%)
Post-term	1 (0.9%)
**Birth Complications**	5 (4.7%)
Respiratory Distress	3
Neonatal Jaundice	2
**Family Chronic Illness**	71 (66.4%)
Cardiovascular Disorders	35 (32.7%)
Endocrine Disorders	21 (19.6%)
Malignancy	7 (6.5%)
Other	8 (7.5%)
**Multiple Chronic Conditions in Family**	31 (29.0%)
**Family History of COVID-19 Infection**	92 (86.0%)
Nuclear family	69 (64.5%)
Mother	12 (11.2%)
Father	4 (3.7%)
Sibling	4 (3.7%)
**Symptomatic at Presentation**	97 (90.7%)
**Mean Number of Symptoms**	2.9 ± 1.8
**Common Symptoms**	
Fever	67 (62.6%)
Cough	11 (10.3%)
Myalgia	5 (4.7%)
Anosmia & Ageusia	5 (4.7%)
Other (headache, sore throat, etc.)	7 (6.5%)
**COVID-19 Diagnosis via PCR**	107 (100.0%)
**PCR Test Frequency (mean ± SD)**	1.2 ± 0.5
**Isolation Duration**	
1–6 days	3 (2.8%)
7–14 days	104 (97.2%)
**COVID Medication Use (mean ± SD)**	2 ± 0.1
**COVID-19-Related Complications**	38 (35.5%)
Taste loss	31 (29.0%)
Smell loss	3 (2.8%)
Pulmonary complications	3 (2.8%)
**COVID-Related Death in Family**	16 (15.0%)
**COVID Reinfection**	8 (7.5%)
**Mutation Type Identified**	
Delta	31 (29.0%)
SARS-CoV-2 (non-specified)	5 (4.7%)
Other variants	4 (3.7%)

**Fig. 1:**
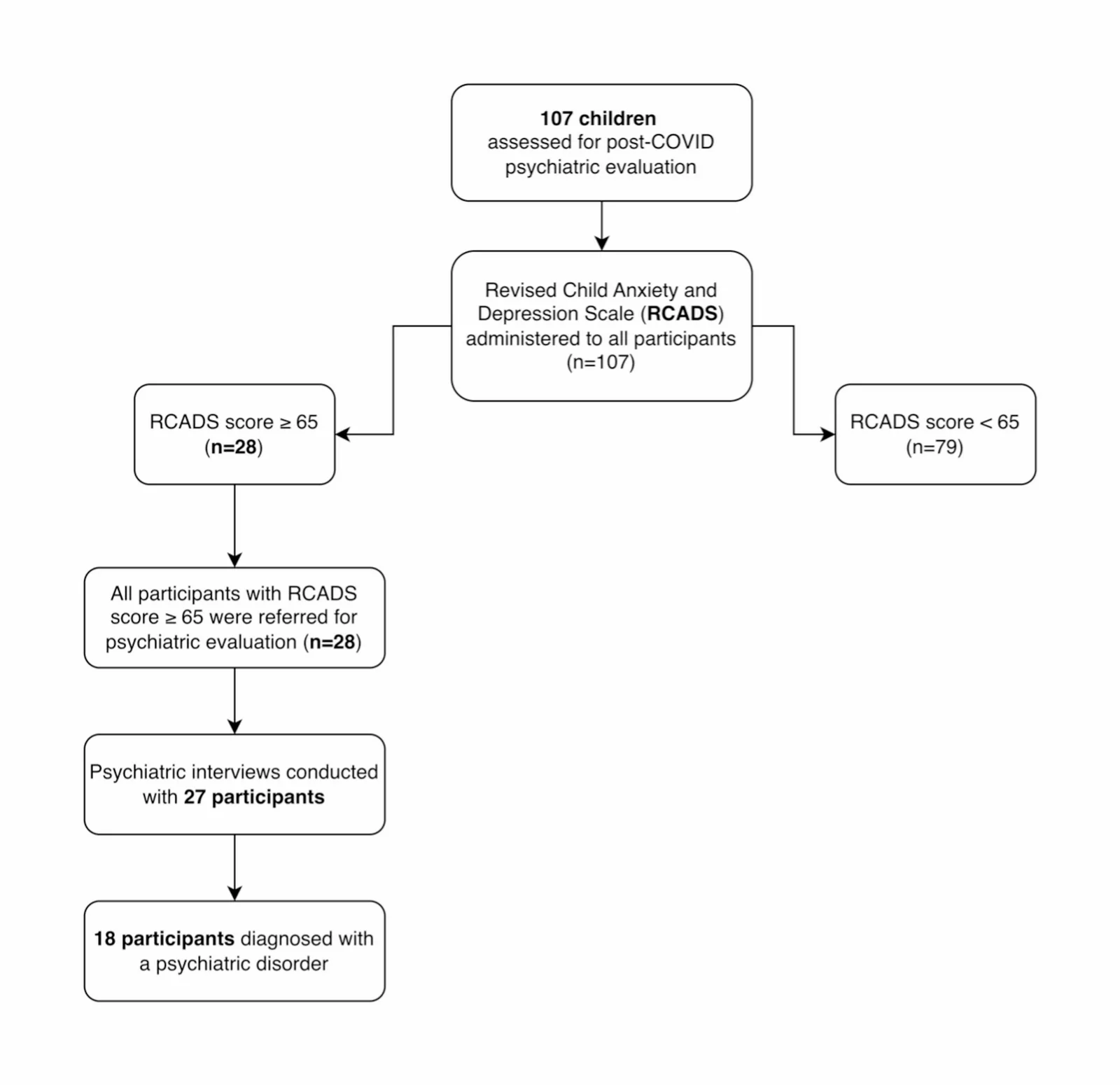
Flowchart of participant recruitment, screening, referral and psychiatric diagnosis. RCADS; Revised Child Anxiety and Depression Scale. All participants (*n* = 107) underwent RCADS assessment following COVID-19 infection. Those with scores ≥ 65 were referred to child and adolescent psychiatry for further evaluation

In the present study, 28 participants (26.2%) scored at or above the clinical threshold (T-score ≥ 65) on the RCADS and were subsequently referred for comprehensive psychiatric evaluation. Of these, 27 completed clinical interviews, and 18 individuals (66.7%) received at least one formal psychiatric diagnosis. The remaining 79 participants (73.8%) scored below the clinical cut-off and were not referred for further psychiatric assessment. The flow of participant recruitment, screening, referral, and diagnostic outcomes is illustrated in Fig. 1.

**Fig. 2:**
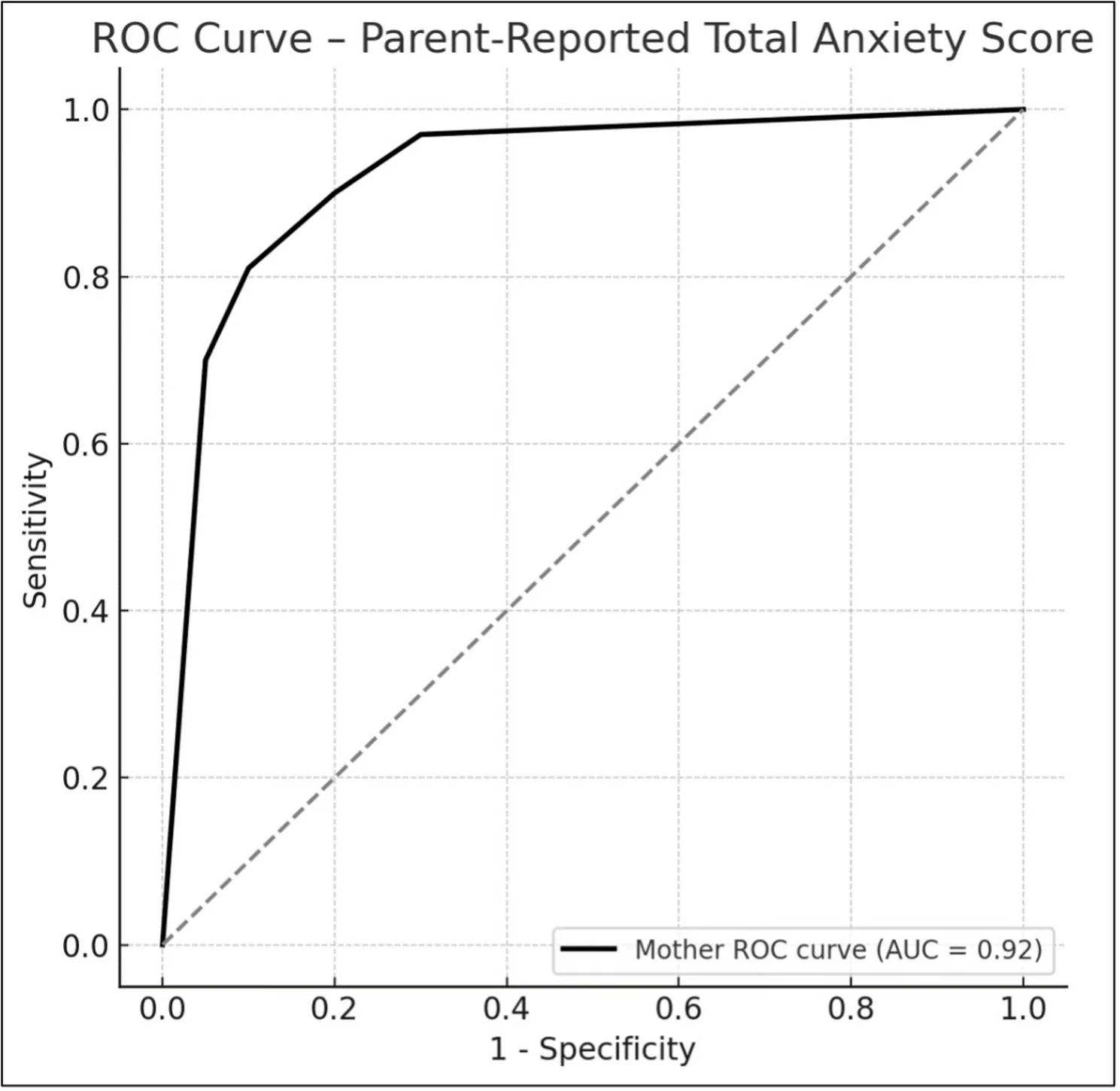
ROC Curve for Parent-Reported Total Anxiety Score (RCADS-Parent). AUC = 0.92, p < 0.001. Cut-off score: 60; Sensitivity: 81.5%, Specificity: 93.8%. ROC; receiver operating characteristic, AUC; area under the curve

**Fig. 3:**
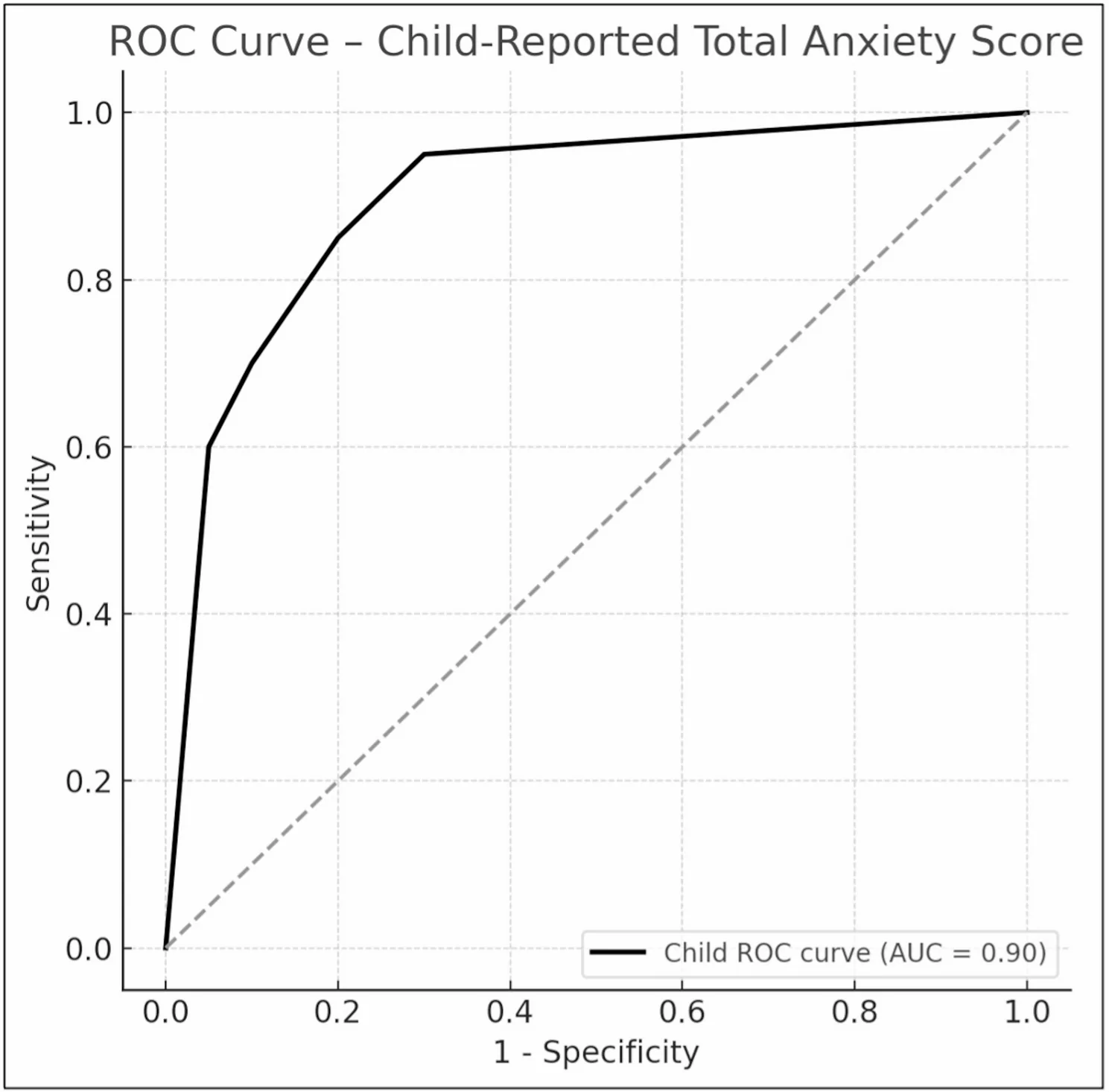
ROC Curve for Child-Reported Total Anxiety Score (RCADS-Child). AUC = 0.90, p < 0.001. Cut-off score: 65; Sensitivity: 70.4%, Specificity: 93.8%. ROC; receiver operating characteristic, AUC; area under the curve

### Pre-COVID-19 psychiatric history

Of the total participants (*n* = 107), 48 children (44.86%) were reported to have at least one psychiatric symptom prior to their COVID-19 infection. Common complaints included attention problems (*n* = 21), irritability or anger (*n* = 18), sleep disturbances (*n* = 10), appetite changes (*n* = 7), anxiety (*n* = 7), depressive symptoms (*n* = 2), and learning difficulties (*n* = 1). Notably, 18 participants reported more than one symptom.

Formal psychiatric evaluation had been conducted in 20 participants (18.69%) prior to infection, and 16 of them (14.95% of the total sample) had received a confirmed psychiatric diagnosis. The most frequent diagnosis was attention-deficit/hyperactivity disorder (ADHD), observed in 13 individuals (81.25% of diagnosed cases). Other diagnoses included anxiety disorder, specific learning disorder, and depressive disorder (Table 2).

**Table 2:** Pre-COVID-19 psychiatric consultations and diagnoses

Category	*n* (%)
**No prior consultation**	87 (81.3%)
**Prior psychiatric consultation**	20 (18.7%)
**No psychiatric diagnosis**	91 (85.0%)
**Prior psychiatric diagnosis**	16 (15.0%)
ADHD	13 (81.3%)
Anxiety disorder	1 (6.5%)
SLD	1 (6.5%)
Other	1 (6.5%)
**Secondary diagnosis (if any)**	
Depression	1 (50.0%)
SLD	1 (50.0%)

Note: Percentages for diagnoses are calculated within the group that received a diagnosis (n = 16)

COVID-19; Coronavirus Disease 2019, SLD; Specific learning disorder, ADHD; attention-deficit/hyperactivity disorder, SLD; Specific learning disorder

### Post-COVID-19 psychiatric symptoms

Following recovery from COVID-19, 60 participants (56.0%) reported experiencing at least one new psychiatric symptom. The most commonly reported complaints included attention problems (*n* = 31), irritability or anger (*n* = 25), sleep disturbances (*n* = 14), appetite changes (*n* = 8), anxiety (*n* = 7), obsessive thoughts (*n* = 3), and depressive symptoms (*n* = 3). Notably, several children reported multiple co-occurring symptoms. When stratified by symptom type, attention problems were the most frequent complaint, followed by irritability and sleep disturbances. Table 3 provides a summary of post-COVID-19 psychiatric symptom prevalence.

**Table 3:** Psychiatric symptoms reported after COVID-19 infection

Symptom Type	*n* (%)
No psychiatric complaint	47 (43.93%)
Any psychiatric complaint	60 (56.07%)
**Symptom Breakdown**	
* Attention problems*	31 (28.97%)
* Irritability/anger*	25 (23.36%)
* Sleep disturbances*	14 (13.08%)
* Appetite changes*	8 (7.48%)
* Anxiety*	7 (6.54%)
* Obsessive thoughts*	3 (2.80%)
* Depressive symptoms*	3 (2.80%)

Note: Percentages are based on the total sample size (N = 107). Multiple symptoms could be reported per participant. COVID-19; Coronavirus Disease 2019

### Post-COVID-19 psychiatric symptoms in previously asymptomatic children and psychiatric referrals

Among the 23 participants with no prior psychiatric complaints, 12 (52.2%) developed a single psychiatric symptom after COVID-19, while 11 (47.8%) exhibited two distinct symptoms. The most common newly developed symptoms in this subgroup were attention problems (*n* = 12), irritability (*n* = 8), depressive mood (*n* = 3), anxiety (*n* = 3), appetite loss (*n* = 2), and obsessive thoughts (*n* = 1).

A total of 14 participants (13.1% of the full sample) sought psychiatric consultation due to post-COVID-19 psychological complaints. Of these, 8 individuals (57.1%) received at least one formal psychiatric diagnosis. Two of the diagnosed participants were identified with comorbid conditions.

The most frequently assigned diagnoses were attention-deficit/hyperactivity disorder (*n* = 3), anxiety disorders (*n* = 3), specific learning disorder (*n* = 2), and obsessive–compulsive disorder (*n* = 2).

Furthermore, a positive family history of psychiatric disorders was reported in 38 participants (35.5%). Depression was the most frequently reported diagnosis among first-degree relatives (*n* = 16), followed by anxiety disorders (*n* = 9), psychotic disorders (*n* = 5), obsessive–compulsive disorder (*n* = 2), behavioral disorders (*n* = 2), and other conditions (*n* = 4) (Table 4).

**Table 4:** Family history of psychiatric disorders among participants

Family Psychiatric History	*n* (%)
Reported	38 (35.5%)
Not reported	69 (64.5%)
**Diagnosis Type (among those reported)**	
* Depression*	16 (42.1%)
* Anxiety disorders*	9 (23.7%)
* Psychotic disorders*	5 (13.2%)
* Obsessive–compulsive disorder*	2 (5.3%)
* Conduct or behavioral disorders*	2 (5.3%)
* Other*	4 (10.5%)
* ADHD*	0 (0%)

Note: Percentages under diagnoses are based on the group with a positive family psychiatric history (n = 38).

ADHD; attention-deficit/hyperactivity disorder

### RCADS scores, clinical interviews, and associated risk factors

A total of 28 participants (26.2%) had RCADS total anxiety and depression T-scores equal to or above 65, indicating clinically significant symptom levels. Among these, 27 individuals underwent follow-up psychiatric interviews. Of those assessed, 18 participants (66.7%) were diagnosed with at least one psychiatric disorder, and 6 individuals (22.2%) were found to have comorbid conditions.

The most frequently identified disorders included generalized anxiety disorder (GAD, *n* = 9), attention-deficit/hyperactivity disorder (ADHD, *n* = 7), and social anxiety disorder (SAD, *n* = 3). Additional diagnoses included depressive disorder (*n* = 2), disruptive mood dysregulation disorder (DMDD, *n* = 1), obsessive–compulsive disorder (OCD, *n* = 1), and oppositional defiant disorder (ODD, *n* = 1).

Participants were stratified into two groups based on whether their RCADS scores (reported by either parent or child) were ≥ 65 (high-score group) or < 65 (low-score group). The relationship between psychiatric background and sociodemographic/clinical variables across these two groups is presented in Table 4.

Statistically significant associations were found between high RCADS scores and (1) a history of birth complications (*p* = 0.010), (2) prior psychiatric complaints (*p* = 0.049), and (3) post-COVID psychiatric referral (*p* = 0.030). Furthermore, those with no prior psychiatric symptoms or consultations were significantly more likely to belong to the low-score group (*p* = 0.001) (Table 5).

**Table 5:** Comparison of clinical variables based on RCADS score groupings

Variable	RCADS ≥ 65 (*n* = 28)	RCADS < 65 (*n* = 79)	*P* value
Gender (female)	19 (67.9%)	43 (54.4%)	0.216
Chronic medical condition	5 (17.9%)	12 (15.2%)	0.740
History of surgery	9 (32.1%)	14 (17.7%)	0.110
Preterm birth	6 (21.4%)	5 (6.3%)	0.081
Birth complications	4 (14.3%)	1 (1.3%)	0.010*
Family history of COVID-19	24 (85.7%)	68 (86.1%)	0.962
Psychiatric complaint at admission	25 (89.3%)	72 (91.1%)	0.772
Post-COVID psychiatric complaint	20 (71.4%)	40 (50.6%)	0.057
Post-COVID psychiatric referral	7 (25.0%)	7 (8.9%)	0.030*
Post-COVID psychiatric diagnosis	4 (14.3%)	4 (5.1%)	0.111
Prior psychiatric complaints	17 (60.7%)	31 (39.2%)	0.049*
Prior psychiatric consultation	7 (25.0%)	13 (16.5%)	0.319
Prior psychiatric diagnosis	6 (21.4%)	10 (12.7%)	0.263
Family history of psychiatric disorder	11 (39.3%)	27 (34.2%)	0.627
No prior psychiatric symptoms/referrals	1 (3.6%)	47 (59.5%)	0.001*
COVID-19-related complications	12 (42.86)	26 (32.91)	0.345

Note: Values are shown as n (%). The Pearson chi-square test was used, and significant p-values are marked with *

RCADS;Revised Child Anxiety and Depression Scale, COVID-19; Coronavirus Disease 2019

### RCADS subscale scores across groups

Statistical comparison of RCADS subscale scores between participants with high (≥ 65) and low (< 65) total scores revealed significant differences across all subdomains, in both parent- and child-reported forms. These findings support the sensitivity of the RCADS in detecting diverse anxiety and depressive symptoms post-COVID-19 (Table 6).

**Table 6:** RCADS subscale scores by group (parent- and child-reported)

Subscale scores (Mean ± SD)	RCADS ≥ 65	RCADS < 65	*p*-value
**Parent-reported RCADS**			
Separation Anxiety	65.1 ± 14.4	48.5 ± 10.5	0.001*
Generalized Anxiety	64.5 ± 10.3	47.4 ± 7.8	0.001*
Panic Disorder	67.7 ± 12.9	50.1 ± 9.7	0.001*
Social Phobia	62.8 ± 12.8	42.8 ± 9.4	0.001*
Obsessive–Compulsive Symptoms	67.3 ± 11.0	50.8 ± 9.1	0.001*
Depression	69.6 ± 12.2	52.3 ± 10.3	0.001*
Total Anxiety	69.3 ± 11.4	46.5 ± 7.9	0.001*
Total Anxiety + Depression	70.3 ± 10.8	47.6 ± 8.4	0.001*
**Child-reported RCADS**			
Separation Anxiety	62.3 ± 12.9	46.3 ± 7.7	0.001*
Generalized Anxiety	57.9 ± 11.9	43.6 ± 8.9	0.001*
Panic Disorder	62.3 ± 12.9	46.5 ± 8.9	0.001*
Social Phobia	59.6 ± 11.8	41.1 ± 10.1	0.001*
Obsessive–Compulsive Symptoms	58.6 ± 14.6	44.1 ± 9.4	0.001*
Depression	64.4 ± 11.2	45.3 ± 11.3	0.001*
Total Anxiety	63.1 ± 12.4	42.2 ± 8.7	0.001*
Total Anxiety + Depression	64.4 ± 12.1	42.4 ± 9.1	0.001*

Note: Independent samples t-test was used and all differences are statistically significant at p < 0.05

RCADS;Revised Child Anxiety and Depression Scale, SD; Standard deviation

### Stratified RCADS score groups and associated variables

Participants were further stratified into three groups based on their total RCADS scores: high (≥ 65), moderate (40–64), and low (< 40). A total of 28 individuals (26.2%) fell into the high-score group, 66 (61.7%) into the moderate group, and 13 (12.1%) into the low-score group.

A significantly higher proportion of male participants was observed in the low-score group compared to moderate and high groups (76.9% vs. 39.4% and 32.1%, respectively; *p* = 0.020). Additionally, participants with a history of birth complications were more likely to have scores ≥ 65 (*p* = 0.029), whereas those with no post-COVID psychiatric symptoms predominantly belonged to the < 40 group (*p* = 0.015).

These findings suggest that male gender, absence of psychiatric symptoms post-infection, and lack of prior complications may serve as protective indicators for lower anxiety and depression scores (Table 7).

**Table 7:** Distribution of demographic and clinical variables across RCADS score groups

Variable	≥ 65 (*n* = 28)	40–64 (*n* = 66)	< 40 (*n* = 13)	*p*-value
**Gender (male)**	9 (32.1%)	26 (39.4%)	10 (76.9%)	0.020*
**Chronic medical condition**	5 (17.9%)	11 (16.7%)	1 (7.7%)	0.682
**History of surgery**	9 (32.1%)	12 (18.2%)	2 (15.4%)	0.273
**Birth type**				
* Preterm birth*	6 (21.4%)	4 (6.1%)	1 (7.7%)	0.224
* Term birth*	22 (78.6%)	61 (92.4%)	12 (92.3%)	
* Post-term birth*	0 (0.0%)	1 (1.5%)	0 (0.0%)	
**Birth complications**	4 (14.3%)	1 (1.5%)	0 (0.0%)	0.029*
**Family history of COVID-19**	24 (85.7%)	55 (83.3%)	13 (100.0%)	0.286
* COVID-positive mother*	4 (16.7%)	8 (14.6%)	0 (0.0%)	0.555
* COVID-positive father*	1 (4.2%)	3 (5.5%)	0 (0.0%)	
* COVID-positive sibling*	2 (8.3%)	4 (7.3%)	0 (0.0%)	
* Extended family living together*	17 (70.8%)	40 (72.7%)	13 (100.0%)	
**Psychiatric complaint at admission**	25 (89.3%)	59 (89.4%)	13 (100.0%)	0.466
**Follow-up without hospitalization (7–14 days)**	26 (92.9%)	65 (98.5%)	13 (100.0%)	0.258
**COVID-related complications**	12 (42.9%)	21 (31.8%)	5 (38.5%)	0.576
**COVID reinfection**	2 (7.1%)	6 (9.1%)	0 (0.0%)	0.521
**Pre-COVID psychiatric complaints**	17 (60.7%)	28 (42.4%)	3 (23.1%)	0.064
**Pre-COVID psychiatric consultation**	7 (25.0%)	12 (18.2%)	1 (7.7%)	0.411
**Pre-COVID psychiatric diagnosis**	6 (21.4%)	9 (13.6%)	1 (7.7%)	0.460
**Psychiatric medication use**	1 (3.6%)	0 (0.0%)	0 (0.0%)	0.411
**Post-COVID psychiatric complaint**	20 (71.4%)	37 (56.1%)	3 (23.1%)	0.015*
**Post-COVID psychiatric referral**	7 (25.0%)	7 (10.6%)	0 (0.0%)	0.055
**Post-COVID psychiatric diagnosis**	4 (14.3%)	4 (6.1%)	0 (0.0%)	0.210
**Family history of psychiatric disorders**	11 (39.3%)	22 (33.3%)	5 (38.5%)	0.835
**No prior psychiatric complaints or referrals**	1 (3.6%)	37 (56.1%)	10 (76.9%)	0.001*
**COVID-19-related complications**	12 (42.86)	21 (31.82)	5 (38.46)	0.576

Note: Pearson chi-square test was used and significant differences at p < 0.05 were shown in bold with *

RCADS; Revised Child Anxiety and Depression Scale, COVID-19; Coronavirus Disease 2019

### Prematurity, birth complications, and parent–child RCADS score discrepancies

Further analysis revealed that participants with total RCADS scores ≥ 65 were significantly more likely to have a history of prematurity (*p* = 0.047), birth complications (*p* = 0.012), and to have received post-COVID psychiatric referrals (*p* = 0.012).

To explore differences between parent and child perceptions of psychiatric symptoms, a subgroup analysis was conducted on participants (*n* = 48) with no prior psychiatric complaints or referrals. In this subgroup, the mean total RCADS score reported by mothers was 48.3 (SD = 10.0), whereas the self-reported child score was substantially lower at 42.2 (SD = 9.5), indicating a statistically and clinically relevant discrepancy in symptom perception (Table 8).

**Table 8:** Total RCADS scores in participants

Group, scores (Mean ± SD)	Parent RCADS	Child RCADS
No prior psychiatric history	48.3 ± 10.0	42.2 ± 9.5
Prior psychiatric history	57.8 ± 14.4	53.1 ± 14.9

RCADS; Revised Child Anxiety and Depression Scale, SD; Standard deviation

### Predictive validity of total anxiety scores (ROC analysis)

To evaluate the predictive value of the RCADS total anxiety subscale in identifying clinical psychiatric cases, receiver operating characteristic (ROC) analyses were performed for both parent and child reports.

The ROC curve for the parent-reported total anxiety score revealed an area under the curve (AUC) of 0.92 (*p* < 0.001), indicating excellent diagnostic accuracy. The optimal cut-off score was determined to be 60, which yielded a sensitivity of 81.5% and a specificity of 93.8% (Fig. 2).

For the child-reported total anxiety score, the AUC was 0.90 (*p* < 0.001), also demonstrating high diagnostic performance. The optimal cut-off score was 65, with 70.4% sensitivity and 93.8% specificity (Fig. 3).

## Discussion

This study investigated post-infectious psychiatric symptoms and diagnoses in children and adolescents following COVID-19 infection. Our findings indicate an increase in psychiatric complaints—especially attentional difficulties and anger problems—following COVID-19 infection. However, despite the heightened symptom burden, the rate of psychiatric service utilization post-COVID remained low compared to pre-pandemic levels. In addition, higher scores on anxiety and depression scales were significantly associated with male gender, prematurity, birth complications, and prior psychiatric history. These findings highlight the complex and lasting psychological consequences of the pandemic on youth mental health.

Our data reveal that common post-COVID psychiatric symptoms included attentional problems, irritability, sleep disturbances, appetite changes, depressive symptoms, and anxiety. These findings are broadly consistent with elevated psychiatric symptom rates documented among adolescents during the pandemic period, even in the absence of confirmed infection. For instance, Chi et al. (2021) [4] reported depression, anxiety, and insomnia rates of 48.2%, 36.7%, and 37.8% respectively among Chinese adolescents during the pandemic. Similarly, Zhou et al. (2020) [26] found a 23.2% prevalence of insomnia, strongly associated with anxiety and depressive symptoms, during the COVID-19 epidemic period. While these studies reflect pandemic-related psychosocial burden broadly, our findings extend this picture by focusing specifically on children with confirmed SARS-CoV-2 infection, suggesting that direct viral exposure may confer additional psychiatric risk beyond the general pandemic context. In a more recent meta-analysis, Hassan et al. (2023) [19] concluded that children who had contracted COVID-19 exhibited significantly higher rates of anxiety, depression, and appetite problems compared to those who had not been infected, possibly related to long-COVID symptomatology. While cognitive symptoms such as concentration problems have been documented, a meta-analysis by Knapp et al. (2024) [13] noted no significant difference in cognitive outcomes, such as attention or memory, between infected and non-infected children. These findings are also consistent with a broader body of evidence linking infectious illness in general to subsequent psychiatric morbidity in children and adolescents. Large-scale nationwide cohort studies conducted prior to the COVID-19 pandemic have demonstrated that treated infections in childhood are associated with a significantly increased risk of a wide range of mental disorders, suggesting that infection-triggered immune dysregulation may represent a shared pathophysiological pathway not specific to COVID-19 [2, 14].

While our findings demonstrate an increased frequency of psychiatric symptoms following COVID-19 infection, these outcomes may also be influenced by pandemic-related contextual factors, such as school closures, social isolation, and family stress, as suggested in the broader literature [8, 18]. However, these variables were not directly assessed in the present study, and the absence of a non-infected control group limits causal interpretation. Therefore, these associations should be interpreted with caution [9, 20].

Despite the observed increase in psychiatric symptoms post-COVID, our study revealed a paradoxical decline in help-seeking behavior. This discrepancy may be due to multiple pandemic-related barriers, including mobility restrictions, healthcare system overload, and shifting parental priorities. Psychological complaints may have been deprioritized amid more pressing somatic concerns. This aligns with previous reports emphasizing decreased psychiatric service utilization during the early pandemic phase [17, 23]. In light of these challenges, scalable and accessible mental health interventions—particularly telepsychiatry—must be integrated into health policy frameworks for future pandemics or similar large-scale disruptions.

In our sample, the most frequently diagnosed disorder post-pandemic was anxiety, consistent with large-scale studies such as the German cohort by Kostev et al. (2023) [15], which showed elevated rates of anxiety and somatoform disorders following COVID-19. Similarly, ADHD was another frequently diagnosed disorder in our cohort. In a Finnish cohort, Auro et al. (2024) [1] observed a post-pandemic increase in ADHD diagnoses without a corresponding rise in pharmacological treatment. These results may reflect a shift in parent or teacher attention to attentional difficulties during online learning, combined with reduced environmental structure.

Another notable finding was that total RCADS scores were significantly lower in children without prior psychiatric complaints or referrals, and even lower among those who reported no psychiatric symptoms post-COVID. This supports the discriminant validity of the RCADS as a screening tool. Furthermore, birth complications were significantly associated with higher RCADS scores (≥ 65), aligning with studies showing that early perinatal adversity may increase susceptibility to later psychiatric problems [10]. These findings support the inclusion of early developmental history in mental health risk screening frameworks.

Boys in our study were more likely to score below 40 on total RCADS, which is consistent with gender-based differences in anxiety and depression symptomatology [21] Additionally, parents—especially mothers—tended to report higher levels of symptoms than their children, even in those with no prior psychiatric history. This is congruent with previous research highlighting moderate concordance between parent and child reports, with caregivers often overestimating internalizing symptoms [7, 12]. The ROC analyses further demonstrated the diagnostic utility of the RCADS, with cut-off points of 60 and 65 for parent- and child-reported total anxiety scores, respectively. These differences reinforce the importance of incorporating multi-informant data when conducting psychiatric assessments in pediatric populations.

### Strengths and limitations

This study’s strengths include face-to-face psychiatric interviews conducted by child and adolescent psychiatrists and the combined use of both parent- and self-report RCADS forms. The use of validated diagnostic thresholds and a structured referral process enhances the methodological rigor.

Nonetheless, several limitations warrant consideration. Although the study involved two clinical centers, the sample was derived from a single regional healthcare network, which may limit generalizability to other settings. Self-report instruments may be prone to reporting bias, especially in younger children with limited emotional vocabulary. Children with intellectual disability or severe sensory impairments were excluded from the study; therefore, the findings may not be generalizable to these potentially more vulnerable groups. In addition, the retrospective nature of the study precludes causal inferences. Furthermore, the absence of a non-infected control group limits our ability to directly compare outcomes and attribute findings specifically to COVID-19 infection.

## Conclusion

This study indicates that psychiatric symptoms—including attentional difficulties, irritability, anxiety, and depressive features—were more frequently reported among children and adolescents following COVID-19 infection. during the COVID-19 pandemic. Despite the rise in symptom burden, psychiatric service utilization appeared limited, suggesting a potential mismatch between clinical need and access to care during public health crises.

Early life factors such as prematurity and birth complications, alongside gender and prior psychiatric history, were associated with heightened vulnerability. The significant relationships between RCADS scores, clinical complaints, and psychiatric diagnoses support the usefulness of this scale as a screening tool in pandemic-related contexts.

These findings highlight the importance of targeted mental health strategies for high-risk groups and highlight the value of incorporating both parent and child perspectives in assessment. Strengthening telepsychiatry capacity, prioritizing mental health within public health planning, and implementing early identification protocols using validated screening instruments may help mitigate long-term psychological impacts in future emergencies. Integrating scalable mental health interventions into routine pediatric care could further improve outcomes.

## Future directions

Future research should adopt longitudinal, multi-center designs with larger samples, comparing pre- and post-pandemic mental health trajectories in youth. Such studies could provide insight into long-term impacts, delineate high-risk groups, and inform targeted early intervention strategies. Inclusion of neurodevelopmental and psychosocial variables such as trauma history, socioeconomic status, and school functioning would further enrich clinical applicability.

## Data Availability

The datasets used and/or analyzed during the current study are available from the corresponding author on reasonable request.
